# From long-term sickness absence to disability retirement: diagnostic and occupational class differences within the working-age Finnish population

**DOI:** 10.1186/s12889-020-09158-7

**Published:** 2020-07-08

**Authors:** Laura Salonen, Jenni Blomgren, Mikko Laaksonen

**Affiliations:** 1 Department of Social Research, University of Turku, Turku, Finland; 2 The Social Insurance Institution of Finland, Helsinki, Finland; 3 Finnish Centre for Pensions, Helsinki, Finland

**Keywords:** Sickness absence, Disability retirement, Occupational class, Diagnosis

## Abstract

**Background:**

It is well documented that sickness absence is strongly associated with disability retirement. A long-term sickness absence (LTSA) in particular increases the risk of disability retirement, but little is known about the variation of this risk across diagnostic causes. Further, as occupational classes differ in their diagnostic profiles, it is likely that the role of diagnosis in the pathway from LTSA to disability retirement varies between occupational classes. We examined how LTSA of different diagnostic causes predicts all-cause disability retirement and disability retirement due to the same diagnostic group or due to some other diagnostic group than that which caused the LTSA spell in different occupational classes.

**Methods:**

Cox proportional hazards models were used to analyse a 70% random sample of all employed Finns aged 25–62 Finns in 2006 (*N* = 1,458,288). Disability retirement was followed from 2007 to 2014. The risk of disability retirement was compared between occupational classes with at least one LTSA spell due to musculoskeletal diseases, mental disorders, respiratory diseases, or circulatory diseases and those who had no LTSA spells due to these diagnostic groups during 2005.

**Results:**

Those who had LTSA due to musculoskeletal diseases or mental disorders transferred more often to disability retirement due to same diagnostic group, whereas those who had LTSA due to respiratory or circulatory diseases transferred more often to disability retirement due to some other diagnostic group. The largest occupational class differences in all-cause disability retirement were found among those with LTSA due to mental disorders. For men, the hazard ratios (HR) varied from HR 5.70 (95% confidence interval (CI) 5.00–6.52) in upper non-manual employees to 2.70 (95% CI 2.50–2.92) in manual workers. For women, the corresponding HRs were 3.74 (95% CI 3.37–4.14) in upper non-manual employees and 2.32 (95% 2.17–2.50) in manual workers.

**Conclusions:**

The association between LTSA and disability retirement varies between diagnostic groups, and the strength of this association further depends on the person’s occupational class and gender.

## Background

Every year, work disability drives a large number of working-age people to exit the labour market due to disability retirement [1], particularly those with long-term sickness absence (LTSA) [2, 3]. However, some illnesses that often cause sickness absenteeism, such as the common cold or minor injuries, do not necessarily cause long-term work disability [4, 5]. In contrast, sickness absence due to mental diagnoses, circulatory diagnoses, musculoskeletal diseases, gastrointestinal diagnoses [5–7], stress-related mental disorders [8], and diseases of the nervous system [4] strongly predicts disability retirement. Results regarding the effects of respiratory diagnoses have been inconsistent [4–6, 9]. Since people can simultaneously have multiple diseases or disorders that restrict their work capacity and additional conditions can develop during a sickness absence spell, a disability pension can be granted due to a different diagnosis than that of the preceding sickness absence. For example, sickness absence due to musculoskeletal diseases has been found to increase the risk of disability retirement due to cancer, mental diseases, and circulatory diseases [7, 10].

However, only few studies have simultaneously examined the role of the diagnosis and occupational classes in the transition from sickness absence to disability retirement. Different occupations permit working with different work ability limitations. For example, musculoskeletal diseases do not necessarily hinder non-manual employees from performing occupational tasks but would adversely affect manual workers. Hence, diagnosis-specific sickness absence can have a distinctive effect on the risk of disability retirement in different occupational classes. Previous studies on the association between diagnosis-specific sickness absence and disability retirement have mainly treated socio-economic variables as confounding variables [6, 7, 10]. The incidences of diagnosis-specific sickness absence and disability retirement vary between occupational classes, the differences being the largest in musculoskeletal diseases [11, 12]. To our knowledge, only one study has examined whether the diagnosis of sickness absence predicts disability retirement differently in different occupational classes, finding that sickness absence due to musculoskeletal diseases increased the risk of disability retirement more in white-collar workers than in blue-collar workers [13]. However, the study included only two diagnostic groups of sickness absence and only two occupational classes.

As knowledge on this topic is scarce, the aim of this study is to assess how the association between LTSA to disability retirement varies between four diagnostic groups and different occupational classes in men and women. We chose four large diagnostic groups that cause LTSA in Finland (musculoskeletal, mental, respiratory and circulatory diseases) to thoroughly examine the role of the LTSA diagnosis in predicting disability retirement across occupational classes. First, we examined, how LTSA due to the included diagnostic groups is associated with all-cause disability retirement and disability retirement due to the same or due to some other diagnostic group than that recorded in the LTSA spell. Second, we studied whether this association varied between occupational classes. Since gender differences are prevalent in LTSA, in disability retirement and their diagnoses [4, 14], as well as in the structure of occupational classes, all analyses were conducted separately for men and women.

## Methods

### Data and methods

The data were obtained from linked registers of the Social Insurance Institution of Finland (Kela), the Finnish Centre for Pensions (ETK) and Statistics Finland. They consist of a 70% representative random sample of employed non-retired Finns aged 25–62 in 2006 (*n* = 1,458,288). The people were followed from 2007 to 2014. Information on socio-demographic characteristics was measured in 2006 and LTSA spells in 2005.

### Measurement of LTSA

LTSA was measured through the receipt of sickness allowance. In Finland, sickness allowance can be paid to non-retired individuals (including also, for example, the unemployed) aged 16–67 to compensate for a short-term loss of income caused by work incapacity, up to approximately 1 year. The first 10 consecutive working days missed due to work incapacity are covered by the employer, and sickness allowance can be paid after this waiting period (Sundays and midweek holidays are not counted as working days) [15]. Medical certification is required for sickness allowance to be provided. In case the work disability continues after 1 year, a disability pension may be granted.

Sickness allowance data were derived from Kela’s register. All LTSA spells that began between January 1, 2005 and December 31, 2005 were included in the study. To eliminate confounding by LTSA spells that lead directly to disability retirement and by unfinished spells, the spells that started in 2006 were not assessed, which has also been the convention in previous studies [16]. The diagnostic groups of LTSA were classified according to the international classification of diseases, 10th revision (ICD-10), and four large diagnostic groups were resultantly extracted. These groups were musculoskeletal diseases (M00–M99), mental and behavioural disorders (F00–F99), diseases of the respiratory system (J00–J99), and diseases of the circulatory system (I00–I99). These were the four largest disease groups in terms of new sickness allowance spells in 2005 covering almost 60% of all new sickness absence spells [17]. Individuals could thus have multiple LTSA spells during the year. 5.5% (*N* = 3630) of men and 7.07% of women (*N* = 6652) who had sickness allowance spells in 2005 had more than one spell that began during that year.

For each participant, sickness absence measures were constructed for the selected diagnostic groups by examining whether the person had a new LTSA spell due to those diagnostic groups. Thus, LTSA was measured as binary variables, where 1 represents having LTSA due to the defined diagnostic group, and 0 indicates not having LTSA due to that diagnostic group. Thus, for example, the reference category for those with LTSA due musculoskeletal diseases consisted of those who did not have LTSA and those who had LTSA only due to some other diagnostic group.

### Measurement of disability retirement

In Finland, all permanent residents are covered by either the national disability pension scheme, which covers individuals aged 16–64, or the earnings-related scheme, which covers individuals aged 18–62. To be granted a full disability pension, a person’s work ability has to be decreased at least to 40%, whereas a decrease to 60% qualifies them for partial disability pension. The applicant’s age, education, occupation and place of residence are taken into account along with the medical assessment when determining eligibility for a disability pension [18]. The eligibility is decided by insurance physicians and specialists belonging to Kela and/or of earnings-related pension insurers, depending on the level of the pension.

Data on disability retirement in this study were retrieved from the registers of ETK (earnings-related pension scheme) and Kela (national pension scheme). Disability retirement included all full and partial disability retirees whose pensions commenced between January 1, 2007 and December 31, 2014. Only the first disability pension that started during the follow-up was taken into account. The diagnostic group of disability retirement was classified as being the same group (e.g. M chapter of ICD-10) or being some other diagnostic group than the one recorded for the sickness absence spell.

### Measurement of occupational class

Information on the occupational class at the end of 2006 was drawn from the register of Statistics Finland. Our variable is based on Statistics Finland’s classification of socio-economic groups, which takes into account the person’s stage in life, occupation and occupational status as well as the nature of the occupation and work [19]. In this study, only the employed population was included. Those included were divided into the following categories: upper non-manual employees, lower non-manual employees, manual workers and self-employed. Those outside employment (retirees, long-term unemployed, students and those with missing or unknown occupational class) were not included (*n* = 269,356). The final number of people analyzed was 1,458,288.

### Other covariates

All analyses were conducted separately for men and women and adjusted for age, marital status and level of urbanization of the home municipality at the end of 2006 (see Table 1 categories of these variables). Information on gender, age, marital status and level of urbanisation were drawn from the population data file of Kela. Age was categorized into four groups in 10-year intervals. Meanwhile, marital status and the level of urbanisation were categorized into three categories in accordance with the classifications of Statistics Finland [20].

**Table 1:** Characteristic of the study population. LTSA diagnostic groups in 2005 and DR in 2007–2014

		Men			Women		
		Distr. (%)	LTSA in 2005 (%)	New DR in 2007–2014 (%)	Distr. (%)	LTSA in 2005 (%)	New DR in 2007–2014 (%)
Occupational class	Upper non-manual employees	23.2	5.3	2.6	21.7	9.2	3.3
	Lower non-manual employees	22.0	8.1	4.3	49.1	13.6	6.5
	Manual workers	41.4	12.2	8.1	21.0	16.9	10.6
	Self-employed	14.5	7.0	7.1	8.2	9.3	7.3
Age	25–34	25.1	5.9	1.6	23.6	8.7	1.9
	35–44	28.8	8.5	3.1	28.2	11.7	3.7
	45–54	29.0	10.7	9.9	30.3	15.1	10.7
	55–62	17.2	11.5	10.2	18.0	17.0	11.2
Marital status	Single	32.7	7.4	4.3	26.4	9.8	4.1
	Married^a^	56.0	9.3	6.1	57.5	13.1	7.0
	Other^b^	11.3	12.0	9.7	16.1	17.6	10.2
Level of urbanization	Urban	56.5	8.5	5.2	58.7	12.6	6.2
	Densely populated	16.5	9.5	6.6	15.9	13.4	7.2
	Rural	26.9	9.7	6.9	25.4	13.7	7.6
Diagnostic group of	*Any LTSA*	*9.0*	*100.0*	*15.2*	*13.0*	*100.0*	*16.4*
LTSA in 2005 ^c^	Musculoskeletal diseases	3.3	100.0	18.7	4.5	100.0	21.5
	Mental disorders	1.0	100.0	18.5	2.3	100.0	18.4
	Respiratory diseases	0.5	100.0	13.3	0.8	100.0	16.7
	Circulatory diseases	0.6	100.0	18.8	0.7	100.0	15.6
Total (%)		100.0	9.0	5.9	100.0	13.0	6.7
N		733,452	66,022	43,222	724,836	94,066	48,851

*LTSA* Long-term sickness absence. *DR* Disability retirement

^a^Including registered partnerships

^b^Including separated, divorced, widowed (from marriage or registered partnership) and those with missing information

^c^Can sum up to over 100% as the same persons can have more than one LTSA spell

### Statistical methods

Cox proportional hazards models were used for data analysis. Each person in the study population was followed from January 1, 2007 until either the disability retirement event or the end of the study period on December 31, 2014. People were censored at the date of the other type of the retirement, or when they turned 63 or died, if either of the latter occurred before the end of the follow-up. Results are presented as hazard ratios (HR) of disability retirement with 95% confidence intervals (CI). In the adjusted models, age, marital status and the level of urbanization of the home municipality will be referred to as demographic variables.

The HRs for all-cause disability retirement and for disability retirement due to the same and due to a different diagnostic group than that of the LTSA were calculated separately for men and women. The models were first run without occupational class and then were subsequently stratified by occupational class. To test whether the LTSA diagnostic group predicts disability retirement differently in different occupational classes, we introduced an interaction term between the LTSA diagnostic group and the occupational class. The analyses were conducted using Stata 15.1. Software.

## Results

9% of the men and 13% of the women had at least one new LTSA spell that started in 2005. During the 8-year follow-up period, 5.9% of the men and 6.7% of the women transferred to disability retirement (*n* = 92,073) (Table 1). The proportion of those transferring to disability retirement was higher among manual workers, older aged workers, those who reported their marital status group as “other” and those who lived in rural municipalities. 15.2% of the men and 16.4% of the women who had at least one LTSA spell transferred to disability retirement. For comparison, around 5.0% of the men and the women who had no LTSA spells in 2005 transferred to disability retirement during the follow-up period (data not shown).

Among men, the proportion of new all-cause disability retirements varied between 13.3% after an LTSA due to respiratory diseases and around 18.8% after an LTSA due to the other examined diagnostic groups. Among women, the proportion of transfers to all-cause disability retirement varied between 15.6% after an LTSA due to circulatory or respiratory diseases and 21.5% after an LTSA due to musculoskeletal diseases. The number of observations reported in Appendix.

After an LTSA due to musculoskeletal or mental diagnoses, both men and women were more likely to transfer to disability retirement due to the same diagnostic group than due to some other diagnostic group (Table 2). In contrast, after an LTSA due to respiratory or circulatory diagnoses, disability retirement was most often granted due to some other diagnostic group.

**Table 2:** New diagnosis-specific DRs in 2007–2014 by LTSA diagnostic group, occupational class and sex

		Diagnostic group of disability retirement (%)					
		Men			Women		
**LTSA diagnostic group**	**Occupational class**	All-cause	Same diagnostic group	Other diagnostic group	All-cause	Same diagnostic group	Other diagnostic group
Musculoskeletal diseases	All occupational classes	18.7	11.2	7.5	21.5	13.8	7.7
	Upper non-manual employees	10.4	4.3	6.1	12.2	6.1	6.1
	Lower non-manual employees	14.8	8.5	6.4	20.3	12.5	7.8
	Manual workers	20.3	12.5	7.9	25.4	17.3	8.1
	Self-employed	21.1	12.7	8.4	22.9	15.7	7.2
Mental disorders	All occupational classes	18.5	11.3	7.3	18.4	11.0	7.4
	Upper non-manual employees	15.3	11.6	3.6	12.9	9.5	3.5
	Lower non-manual employees	16.9	11.6	5.3	18.3	10.8	7.5
	Manual workers	19.7	10.1	9.7	23.0	12.3	10.7
	Self-employed	24.6	15.3	9.3	22.5	13.9	8.5
Respiratory diseases	All occupational classes	13.3	1.2	12.1	16.7	1.6	15.2
	Upper non-manual employees	6.7	0.3	6.4	10.5	1.3	9.2
	Lower non-manual employees	10.0	0.6	9.4	15.2	1.3	13.9
	Manual workers	16.7	1.5	15.1	23.9	2.0	21.9
	Self-employed	14.4	1.9	12.5	23.4	3.4	19.9
Circulatory diseases	All occupational classes	18.8	6.0	12.8	15.6	2.3	13.4
	Upper non-manual employees	13.8	5.2	8.6	8.8	1.7	7.1
	Lower non-manual employees	15.1	5.4	9.7	14.9	2.2	12.7
	Manual workers	22.1	6.6	15.4	20.9	2.7	18.2
	Self-employed	18.0	5.7	12.3	16.5	2.3	14.2

*LTSA* Long-term sickness absence, *DR* Disability retirement, The number of observations reported in Appendix

These associations were similar across occupational classes apart from exceptions. In men, upper non-manual employees who had LTSA due to musculoskeletal diseases transferred to disability retirement more often due to some other diagnostic group than due to the same diagnostic group. On the other hand, women, equally often transferred to disability retirement due to the same and due to another diagnostic group. Among both men and women, manual workers with an LTSA due to mental disorders transferred almost as often to disability retirement due to the same and due to some other diagnostic group, unlike other occupational classes.

### LTSA as a predictor of disability retirement among men

Among men, having an LTSA due to mental disorders (HR 3.40) and musculoskeletal diseases (HR 3.14) had the strongest association with all-cause disability retirement compared to those who did not have LTSA due to these diagnostic groups (Table 3). The association with disability retirement due to the same diagnostic group as during the previous LTSA was the strongest among those who had an LTSA due to mental disorders (HR 11.0), followed by those with respiratory diseases (HR 8.70). Mental disorders, respiratory diseases, and circulatory diseases had an equally strong association with disability retirement due to some other diagnostic group. The association was weakest concerning LTSA due to musculoskeletal diseases.

**Table 3:** HRs for LTSA in predicting diagnosis-specific DR by occupational class in men

		Diagnostic group of disability retirement								
		All-cause			Same diagnostic group^1^			Other diagnostic group^2^		
**LTSA diagnostic group**	**Occupational class**	HR	CI	*Sig.*^*3*^	HR	CI	*Sig.*	HR	CI	*Sig.*
Musculoskeletal diseases	All occupational classes	3.14	3.05–3.24		5.30	5.10–5.50		1.61	1.49–1.76	
	Upper non-manual employees	3.58	3.11–4.11	*(ref.)*	8.76	7.00–10.96	*(ref.)*	2.47	2.07–2.96	*(ref.)*
	Lower non-manual employees	3.17	2.00–3.45		6.35	5.63–7.15	**	1.87	1.64–2.12	
	Manual workers	2.46	2.37–2.56	***	3.63	3.45–3.81	***	1.61	1.52–1.71	***
	Self-employed	3.31	3.02–3.62		5.73	5.09–6.47	***	1.91	1.66–2.20	
Mental disorders	All occupational classes	3.40	3.22–3.59		11.00	10.2–11.8		1.92	1.83–2.01	
	Upper non-manual employees	5.70	5.00–6.52	*(ref.)*	13.64	11.65–15.95	*(ref.)*	2.00	1.52–2.58	*(ref.)*
	Lower non-manual employees	4.01	3.57–4.51	***	10.56	9.14–12.20	**	1.68	1.37–2.06	
	Manual workers	2.70	2.50–2.92	***	9.37	8.36–10.49	***	1.52	1.37–1.71	
	Self-employed	4.23	3.62–4.95	**	13.83	11.31–16.91		1.93	1.50–2.47	
Respiratory diseases	All occupational classes	2.33	2.14–2.50		8.70	6.40–11.70		2.16	1.98–2.37	
	Upper non-manual employees	2.66	1.98–3.57	*(ref.)*	10.85	2.62–44.82	*(ref.)*	2.57	1.91–3.45	*(ref.)*
	Lower non-manual employees	2.31	1.85–2.88		8.03	3.28–19.68		2.22	1.77–2.77	
	Manual workers	2.05	1.83–2.28		6.85	4.77–9.82		1.90	1.70–2.13	
	Self-employed	2.34	1.78–3.08		13.15	6.17–28.05		2.00	1.49–2.69	
Circulatory diseases	All occupational classes	2.58	2.40–2.77		6.60	5.80–7.50		1.98	1.81–2.16	
	Upper non-manual employees	4.03	3.29–4.93	*(ref.)*	11.4	8.21–16.06	*(ref.)*	2.95	2.29–3.79	*(ref.)*
	Lower non-manual employees	2.59	2.15–3.11	**	7.15	5.21–9.81		1.86	1.48–2.34	**
	Manual workers	2.20	2.00–2.42	***	5.57	4.69–6.62	***	1.73	1.55–1.93	***
	Self-employed	2.32	1.89–2.85	**	5.46	3.81–7.82	**	1.84	1.45–2.34	**

*LTSA* Long-term sickness absence, *DR* Disability retirement, *HR* Hazard ratio, *CI* Confidence interval

In each cell, the reference group consist of those who had no long-term LTSA due to each diagnostic group per occupational class. All models are adjusted for demographic variables

^1^ Disability retirement due to the same diagnostic group as in previous LTSA spell

^2^ Disability retirement due to some other diagnostic group than in previous LTSA spell

^3^ * *p* < 0.001 ** *p* < 0.02 *** *p* < 0.05. The significance level of the interaction test between occupational class and LTSA diagnostic group

In general, in men, across most diagnostic groups, upper non-manual employees had the highest HR and manual workers had the lowest HR for disability retirement, especially when the LTSA was due to mental disorders. Upper non-manual employees that had an LTSA spell due to mental disorders had an HR of 5.70 (95% CI 5.00–6.52) whereas manual workers had an HR of 2.70 (95% CI 2.50–2.92) for all-cause disability retirement. An LTSA due to circulatory diseases increased the risk of disability retirement more among upper non-manual employees than in other occupational classes. Across occupational classes, the associations were stronger between LTSA and disability retirement due to the same diagnostic group than between LTSA and disability retirement due to some other diagnostic group. Also, occupational class differences in the HRs were larger with respect to associations between LTSA and disability retirement due to the same diagnosis than with disability retirement due to some other diagnostic group.

### LTSA as a predictor of disability retirement among women

An LTSA due to musculoskeletal diseases was the strongest predictor of all-cause disability retirement (HR 3.20) among women (Table 4). Having an LTSA due to respiratory diseases had an especially strong association with disability retirement within the same diagnostic group (HR 19.0). Furthermore, LTSA due to respiratory diseases was the strongest predictor (HR 2.34) while LTSA due to mental disorders was the weakest predictor of disability retirement due to some other diagnostic group.

**Table 4:** HRs for LTSA in predicting diagnosis-specific DR by occupational class in women

		Diagnostic group of disability retirement								
		All-cause			Same diagnostic group^1^			Other diagnostic group^2^		
**LTSA diagnostic group**	**Occupational class**	HR	CI	*Sig.*^*3*^	HR	CI	*Sig.*	HR	CI	*Sig.*
Musculoskeletal diseases	All occupational classes	3.20	3.12–3.28		4.96	4.80–5.13		1.93	1.85–2.01	
	Upper non-manual employees	3.21	2.89–3.58	*(ref.)*	7.50	6.42–8.77	*(ref.)*	2.08	1.80–2.40	*(ref.)*
	Lower non-manual employees	3.17	3.05–3.29		4.90	4.67–5.15	***	2.00	1.88–2.11	
	Manual workers	2.46	2.37–2.57	***	3.32	3.16–3.50	***	1.55	1.4–1.65	***
	Self-employed	3.41	3.07–3.78		5.45	4.80–6.19	**	1.83	1.54–2.19	
Mental disorders	All occupational classes	2.81	2.71–2.92		6.94	6.60–7.30		1.49	1.40–1.57	
	Upper non-manual employees	3.74	3.37–4.14	*(ref.)*	7.34	6.49–8.30	*(ref.)*	1.62	1.34–1.95	*(ref.)*
	Lower non-manual employees	2.90	2.76–3.05	***	6.81	6.36–7.29		1.57	1.46–1.70	
	Manual workers	2.32	2.17–2.50	***	6.52	5.90–7.22	*	1.33	1.20–1.47	
	Self-employed	3.47	2.93–4.11		8.65	6.98–10.72		1.74	1.34–2.27	
Respiratory diseases	All occupational classes	2.57	2.40–2.73		19.00	15.23–23.70		2.34	2.19–2.50	
	Upper non-manual employees	3.14	2.60–3.78	*(ref.)*	32.94	18.39–58.99	*(ref.)*	2.74	2.26–3.35	*(ref.)*
	Lower non-manual employees	2.40	2.18–2.63	*	20.80	14.83–29.15		2.22	2.02–2.44	
	Manual workers	2.36	2.12–2.64	*	12.91	8.67–19.23	*	2.17	1.93–2.43	*
	Self-employed	3.63	2.84–4.64		29.10	15.01–56.40		3.13	2.42–4.06	
Circulatory diseases	All occupational classes	1.96	1.82–2.11		5.81	4.80–7.03		1.75	1.62–1.89	
	Upper non-manual employees	2.15	1.69–2.74	*(ref.)*	8.22	4.70–14.38	*(ref.)*	1.80	1.38–2.36	*(ref.)*
	Lower non-manual employees	1.98	1.79–2.20		6.10	4.65–8.00		1.75	1.57–1.96	
	Manual workers	1.63	1.45–1.85	*	4.27	3.04–6.00	*	1.49	1.31–1.69	
	Self-employed	2.13	1.66–2.73		6.08	3.11–11.87		1.88	1.44–2.46	

*LTSA* Long-term sickness absence, *DR* Disability retirement, *HR* Hazard ratio, *CI* Econfidence interval

In each cell, the reference group consist of those who had no long-term LTSA due to each diagnostic group per occupational class. All models are adjusted for demographic variables

^1^ Disability retirement due to the same diagnostic group as in previous LTSA spell

^2^ Disability retirement due to some other diagnostic group than in previous LTSA spell

^3^ * *p* < 0.001 ** *p* < 0.02 *** *p* < 0.05. The significance level of the interaction test between occupational class and LTSA diagnosis group

Additionally, among the women that had LTSA due to the examined diagnostic groups, upper non-manual employees and self-employed had the highest HRs, whereas manual workers had the lowest HRs of disability retirement. However, the differences between occupational classes were smaller than in men. Also echoing the men’s results, the largest occupational class differences in the risk of all-cause disability retirement were found among those with an LTSA due to mental disorders where upper non-manual employees had an HR of 3.74 (3.37–4.14) and manual workers had an HR of 2.32 (2.17–2.50). Among upper non-manual employees, LTSA due to mental disorders was the strongest predictor of all-cause disability retirement among the four diagnostic groups. Among lower non-manual employees the strongest predictor of all-cause disability retirement was LTSA due to musculoskeletal diseases. Among manual workers and the self-employed, LTSA due to musculoskeletal disorders, mental disorders, and respiratory diseases were equally strong predictors of all-cause disability retirement. No large occupational class differences were found among the diagnostic groups of LTSA with respect to predicting disability retirement due to the same diagnostic group or due to some other diagnostic group.

## Discussion

### Main results

In this study, we examined the association between diagnosis-specific LTSA and all-cause disability retirement, as well as with disability retirement due to the same or due to some other diagnostic group than that recorded in a pre-retirement LTSA spell. Clear differences were found between the LTSA diagnostic groups in predicting disability retirement. The association was the strongest when both the LTSA and disability retirement were due to the same diagnostic group, but significant associations were also found when disability retirement was caused by a different diagnostic group. In general, upper non-manual employees consistently had the highest HRs, whereas manual workers had the lowest HR for disability retirement.

### Diagnostic differences

In general, LTSA due to any of the four diagnostic group was strongly associated with all-cause disability retirement among men and women. LTSA due to musculoskeletal diseases and mental disorders had the strongest associations with all-cause disability retirement, in accordance with previous studies [4–6, 9, 10, 21, 22]. A novel finding was that those with LTSA due to mental disorders and musculoskeletal diseases transferred more often to disability retirement due to the same diagnostic group than due to some other diagnostic group. Additionally, LTSA due these diagnostic groups had slightly stronger association, in terms of HRs, with all-cause disability retirement than LTSA due to respiratory or circulatory diseases. While the differences between LTSA diagnostic groups were small, LTSA due to mental disorders increased the risk of disability retirement slightly more than musculoskeletal diseases in men, whereas in women the order was the opposite. This has also been found in a previous study [9].

A higher proportion of those who had an LTSA due to respiratory or circulatory diseases transferred to disability retirement due to some other diagnostic group. However, in comparison to those who had no LTSA due to these diagnostic causes, having an LTSA was more strongly associated with disability retirement due to the same diagnostic group than due to some other diagnostic group, resembling the results pertaining to mental and musculoskeletal diseases. The results regarding circulatory diseases are in accordance with previous findings [4, 21] while the results regarding respiratory diseases partly corroborate results of previous studies that have found evidence that demonstrated both a strong association [4, 6, 23] and no association [5] between LTSA due to respiratory diseases and disability retirement. The mixed results are probably due to differences in the study population, and/or variance in the definitions of the length of LTSA and that of diagnostic groups. Respiratory diseases vary between subgroups – ranging from a mild cold to chronic obstructive pulmonary disease – across which the risk of disability retirement is likely to vary. As this study only included long-term LTSAs, the respiratory diseases were probably severe. However, as only a relatively small number of individuals had an LTSA and/or disability retirement due to respiratory diseases in this study, especially when stratified by occupational class, caution must be practiced in interpreting the magnitude of this association.

Contrary to previous studies that have studied the association between LTSA and disability retirement, we also included information on the diagnosis of disability retirement, revealing that the diagnostic groups were also associated with disability retirement due to some other diagnostic group. The general mechanisms that govern the association between LTSA and disability retirement due to some other diagnosis than that during the LTSA spell can only be hypothesized in this study. First, a disability retirement can be admitted only due to one main diagnosis. Therefore, in the case of multimorbidity, only one condition can be chosen to be the main diagnosis. Due to a potentially long period between the LTSA measurement and the event of disability retirement in this data set, individuals could have also suffered from other diseases or disorders during the period between the measured LTSA spell and their eventual disability retirement. It is also possible that somatic diseases are recognised first while mental diagnoses remain unreported [24]. Multimorbidity is strongly associated with poor health [25] and work disability [26, 27], rendering people vulnerable to additional illnesses. For example, respiratory diseases are associated with low work ability [28] and poor self-rated health [29]. They also commonly co-occur with diabetes [30] and depressive symptoms [29], and result in an increased risk of disability retirement especially when combined with another chronic disease or depression [31]. These results imply that disability retirement can be preceded by multimorbidity that clinical practitioners should take into account when planning rehabilitation measures.

### Occupational class differences

In comparison to other occupational classes, a higher proportion of manual workers transferred to disability retirement. However, among upper non-manual employees having an LTSA increased the risk of disability retirement the most. On the other hand, this increase in risk as the least among manual workers. Previous studies have found similar results. For example, studies found that upper non-manual employees that have taken sickness allowance have a higher relative risk of disability pension than other occupational classes, especially when the LTSA was due to mental disorders [32], and due to musculoskeletal diseases [13]. Additionally, a French study found that those in a higher occupational position had a higher risk of poor health after sick leave due to cancer and mental disorders compared to those in a lower occupational position [33]. The higher disability retirement risk in upper non-manual employees is related to the relative differences within occupational classes. However, in absolute terms, the risk of disability retirement is the highest among manual workers.

There are several possible explanations for this. Manual workers have typically higher rates of LTSA and disability retirement [11, 12], but, they may more frequently have an LTSA that does not lead to disability retirement. Instead, they may be more likely to be forced out of the workforce (due to unemployment, for example) than non-manual employees [3], their applications for disability retirement are more often rejected [34], or they may need a longer LTSA to recover from illnesses or diseases. Manual workers’ work environment can force them to take sickness absence even if they have less severe work disability. Contrarily, upper non-manual employees’ work environment can provide more flexibility and enables them to continue working even with a disease or disorder. Since the reference groups in this study consisted of those with no LTSA due to the examined diagnostic groups in each occupational class, our results may reflect the fact that despite generally having less work ability problems, upper non-manual employees are more likely to transfer to disability retirement once they avail sickness absence. Their employers may prefer that they remain on sickness absence until fully recovered, as positions in such occupations can be difficult to replace [35]. Further, upper non-manual employees often have psychologically demanding jobs [36, 37], which are especially difficult to return to after mental health problems. More research should be conducted on the possible explanations regarding why upper non-manual employees have a relatively higher risk of disability retirement than manual workers once they undergo LTSA.

Among women, LTSA due to musculoskeletal diseases increased the risk of all-cause disability retirement the most in lower non-manual employees and manual workers. Meanwhile, LTSA due to mental disorders was the strongest predictor of disability retirement among upper non-manual employees and one of the strongest predictors of the same among the self-employed. This is likely to reflect the fact that many women work sectors that are physically demanding such as the health care sector. Among upper non-manual employee men, LTSA due to circulatory diseases had a relatively strong association with disability retirement compared to other occupational classes. According to a previous study, cardiovascular diseases are more strongly associated with disability retirement among those in low occupational classes than in higher occupational classes [38]. However, while the previous study [35] studied the additional and synergistic effects of both occupational class and cardiovascular diseases, we examined the effect of LTSA through stratification according to occupational class. Thus, while manual workers have the highest risk of disability retirement across all occupational classes, having an LTSA due to circulatory diseases increases the risk of disability retirement more among upper non-manual employees.

### Strengths and limitations

The main strength of this study was its ability to utilize a large data set that comprised several combined high-quality national register sources, including a 70% random sample of the Finnish employed population that consisted of almost 1.5 million people. These data ensured an almost complete follow-up of the same individuals with negligible and random missing information and the ability to use date-specific information pertaining to both LTSA and disability retirement. However, the data lacked information on short-term sickness absence (less than 10 days), previous work trajectories, work environment, subjective health, health behaviour and the type of disability pension (i.e. information on part-time or full-time, and permanent or fixed-term disability retirement) which may have explained some of the observed associations. No causal effects could be established due to the observational research setting. Since the analysis was conducted only with Finnish data, whether these results hold in other countries is yet to be ascertained. The results may be generalized with caution to other countries with reasonably similar social security systems with respect to work disability. Future studies should examine the possible explanations for the occupational class differences in the transition from sickness absence to disability retirement due to different diagnoses. Introducing information on factors such as health behaviour and work environment can help to understand why some diagnoses are more important than others in predicting disability retirement, and where the occupational differences in this association emerge. The role of comorbidity in the transition to disability retirement and the occupational class differences in it should be studied more in detail, for example, by adding information on an individual’s medical history. Lastly, the research design used in the current study could be used to examine the role of more specific diagnoses in the transition to disability retirement.

## Conclusions

Among both men and women, LTSA due to mental disorders and musculoskeletal diseases resulted in the greatest increase in the risk of all-cause disability retirement and disability retirement due to the same diagnostic group. Additionally, LTSA due to respiratory diseases was the strongest predictor of disability retirement due to some other diagnostic groups. In general, manual workers had the highest number of new disability retirements across all occupational classes, but an LTSA increased the risk of disability retirement the most among upper non-manual employees. In all, our results confirm that there is a complex interplay in the associations between disability retirement and diagnosis, occupational class and gender. However, the precise mechanism that governs the occupational class differences in the association between LTSA and disability retirement, remains to be elucidated.

## Data Availability

The data that support the findings of this study are available on request from the registered data holders (Kela, ETK, and Statistics Finland). The data are not publicly available due to data protection laws and regulations.

## Appendix

**Table 5:** The number of diagnosis-specific DRs in 2007–2014 by LTSA diagnosis, occupational class and sex

		Diagnosis of disability retirement					
		Men			Women		
**LTSA diagnostic group**	**Occupational class**	All-cause	Same diagnostic group	Other diagnostic group	All-cause	Same diagnostic group	Other diagnostic group
Musculoskeletal diseases	All occupational classes	4524	2699	1825	7007	4499	2508
	Upper non-manual employees	211	87	124	383	191	192
	Lower non-manual employees	574	327	247	3276	2023	1253
	Manual workers	3231	1980	1251	2930	1999	931
	Self-employed	508	305	203	418	286	132
Mental disorders	All occupational classes	1357	825	532	3103	1852	1251
	Upper non-manual employees	235	179	56	418	306	112
	Lower non-manual employees	308	212	96	1700	1007	693
	Manual workers	647	330	317	835	446	389
	Self-employed	167	104	63	150	93	57
Respiratory diseases	All occupational classes	516	45	471	980	91	889
	Upper non-manual employees	46	< 10	44	115	14	101
	Lower non-manual employees	82	< 10	77	472	40	432
	Manual workers	336	31	305	325	27	298
	Self-employed	52	< 10	45	68	10	58
Circulatory diseases	All occupational classes	764	245	519	778	112	666
	Upper non-manual employees	99	37	62	67	13	54
	Lower non-manual employees	115	41	74	379	55	324
	Manual workers	452	136	316	268	35	233
	Self-employed	98	31	67	64	< 10	55

*LTSA* Long-term sickness absence, *DR* Disability retirement

## Abbreviations

LTSA: Long-term sickness absence
DR: Disability retirement
ICD-10: The international classification of diseases, 10th revision
ETK: Finnish Centre for Pensions
Kela: The Social Insurance Institution of Finland
HR: Hazard ratio
CI: Confidence interval
